# Transcriptomic Reprogramming of the Human Placenta Following Maternal Opioid Exposure: Identification of Altered Metabolic and Translational Pathways

**DOI:** 10.3390/cimb48090900

**Published:** 2026-09-03

**Authors:** Po’okela K. Ng, Vedbar S. Khadka, Connor Howe, Jonathan Riel, Men-Jean Lee, Claire E. Kendal-Wright

**Affiliations:** 1 Department of Natural Sciences and Mathematics, Chaminade University of Honolulu, Honolulu, HI 96816, USA; sa209931@atsu.edu; 2 Department of Quantitative Health Sciences, John A. Burns School of Medicine, University of Hawai’i, 651 Ilalo St, Honolulu, HI 96813, USA; vedbar@hawaii.edu; 3 Department of Obstetrics, Gynecology, and Women’s Health, John A. Burns School of Medicine, University of Hawai’i, 1319 Punahou St, Honolulu, HI 96826, USA; chowe96@hawaii.edu (C.H.); jriel@hawaii.edu (J.R.); 4 Department of Obstetrics and Gynecology and Reproductive Health, Rutgers University, 185 South Orange Avenue, MSB E-506, Newark, NJ 07101-1709, USA; menjean.lee@rutgers.edu

**Keywords:** placenta, opioid, RNAseq, Ingenuity Pathway Analysis, transcriptomics, cell stress, Neonatal Opioid Withdrawal Syndrome

## Abstract

Background: Opioid use during pregnancy is linked to preterm birth, fetal growth restriction, and Neonatal Opioid Withdrawal Syndrome. While the placenta is the primary regulator of the uterine environment, the precise molecular mechanisms by which opioids dismantle placental function remain poorly defined. Methods: The transcriptomic landscape of opioid-exposed human placentas from a unique Hawai’i-based cohort was characterized. Results: Transcriptional signatures were defined by genes involved in translational inhibition and metabolic attenuation. Integrative network modeling predicted a prominent stress-signaling hub centered on p38 Mitogen-Activated Protein Kinase and Extracellular Signal-Related Kinase 1/2, paralleling a broad suppression of ribosomal protein transcripts (Ribosomal Protein 27S, -29, and -39). This response was further characterized by the inhibition of the Myelecytomatosis Proto-Oncogene regulatory hub, predicting a loss of mitochondrial phosphate transport through associated genes (via Solute Carrier Family 25A3) and cell-cycle progression (via S-Phase Kinase-Associated Protein 1). Additionally, the genes in this model suggested upregulation of antisense regulatory brakes, such as *RAP2C* Antisense RNA 1, which might further reinforce this inhibitory state. Conclusion: The findings suggest that prenatal opioid exposure is associated with the disruption of placental homeostasis. This may be coordinated by protein synthesis and bioenergetic flux, as inferred from the modeled eukaryotic Translation Initiation Factor-mediated Integrated Stress Response. These findings establish a localized, clinical baseline of human placental stress, highlighting candidate molecular regulatory nodes to guide future mechanistic studies and biomarker discovery in exposed pregnancies.

## 1. Introduction

The ongoing opioid crisis represents a major public health challenge in the United States, disproportionately impacting women of childbearing age, who account for a substantial proportion of individuals with opioid use disorder (OUD) [1]. Consequently, prenatal opioid exposure has become increasingly prevalent, carrying significant risks for adverse perinatal outcomes, including miscarriage, preterm birth, and fetal growth restriction (FGR) [2]. Surviving infants face heightened susceptibility to Neonatal Opioid Withdrawal Syndrome (NOWS), a complex clinical condition characterized by central nervous system hyperirritability, autonomic dysregulation, and gastrointestinal dysfunction [3,4]. While national NOWS incidence estimates a range between 6 and 7 cases per 1000 hospital births, significant regional variation exists, with high-prevalence areas experiencing rates exceeding 30 per 1000 live births, placing a substantial operational and financial burden on neonatal intensive care units [5,6].

A central clinical challenge in managing NOWS is that withdrawal severity does not consistently correlate with maternal opioid dosage or maternal serum drug levels at delivery [7,8]. This clinical discordance suggests that fetal drug exposure and subsequent toxicity are modulated by complex intrauterine dynamics, including drug sequestration in amniotic fluid and variation in placental clearance [9]. Furthermore, observations in twin pregnancies that demonstrate discordant withdrawal severity point to localized placental adaptations and genetic modifiers as primary determinants of fetal outcomes [10].

Far from functioning as a passive barrier, the human placenta is a dynamic organ that regulates fetal growth, immunologic tolerance, and nutrient transfer [11]. Opioid administration alters placental structure and function; preclinical models demonstrate impaired villous vascularization and chronic hypoxia [12,13], while clinical studies link opioid exposure to altered trophoblast differentiation and placental malperfusion [14]. Functional opioid receptors (µ, δ, κ) and Toll-like receptor 4 (TLR4) expressed across human trophoblast lineages regulate localized angiogenesis, cell proliferation, and pro-inflammatory cascades, offering a potential mechanistic bridge between maternal OUD and adverse birth outcomes [15,16,17].

Recent high-throughput transcriptomic investigations have sought to map the molecular networks underlying these placental insults. Preclinical animal models, where drug exposure, timing, and environmental factors are strictly controlled, demonstrate marked alterations in trophoblast differentiation markers and growth factor signaling [18]. Conversely, human clinical studies report broader immunologic dysregulation and altered epigenetic profiles in term tissue [19,20]. However, a notable lack of consensus remains regarding which markers are differentially expressed across prior studies. These discrepancies largely stem from key methodological divergences, including variations in tissue handling, post-mortem RNA degradation, co-exposures (e.g., tobacco, polysubstance use), differences in drug formulations, and population-specific genetic backgrounds.

To resolve this inter-study discordance, high-integrity (RIN > 8.0), fresh-frozen term placental tissue from a demographically aligned, unique Hawai’i-based biospecimen repository was used. This provided a critical opportunity to identify conserved, core molecular pathways of human placental adaptation. Accordingly, this study aimed to characterize opioid-exposed placental transcriptomic reprogramming in this unique human cohort, using unbiased RNA sequencing (RNA-seq) to identify the core transcriptional programs and stress-response networks that underpin adverse neonatal outcomes in Hawai’i’s population.

## 2. Materials and Methods

### 2.1. Retrieval of Archived Frozen Samples

Patient samples exposed (*n* = 10) or not exposed to opioids (*n* = 10) were demographically aligned for parity, maternal age, gestational age at delivery, maternal BMI, ethnicity, mode of delivery, neonatal weight, cigarette smoking (one in each group), preterm labor, and sex of the fetus. Samples were collected from the Hawai’i Biorepository in accordance with its IRB-approved protocol (WIRB Study Number 1107593). The exclusion criteria included fetal growth restriction, diabetes, gestational hypertension, chronic hypertension, preeclampsia (with or without severe features), eclampsia, cocaine use, and hard-liquor use. Six patient samples were selected per group for transcriptomic analysis based on RNA quality (purity and RIN score) and the cohort’s demographic information in order to have the highest-quality starting material.

Statistical differences between the opioid-exposed and non-exposed patient groups were analyzed by demographic variables and tested by Fisher’s exact test. Differences were considered statistically significant if *p* ≤ 0.05.

### 2.2. RNA Isolation

Archived placental specimens were snap-frozen immediately after delivery. Pieces were cut through the full thickness of the placental plate from two lobules taken equidistantly from the site of the cord insertion to the edge of the placenta and stored at −80 °C. However, only one sample was randomly selected per placenta to maintain uniform experimental processing and avoid intra-organ batch variance during library construction. Upon use, they were washed twice in 1× PBS and cut into 30 mg portions from the frozen placenta lobule. Homogenization was completed using a stainless-steel Bio Pulverizer (Lab Supply Network, Atkinson, NH, USA) in liquid nitrogen. RNA was isolated from this now-powdered placenta using RNeasy mini kits with DNase treatment (Qiagen, Germantown, MD, USA), according to the manufacturer’s instructions. RNA concentration and purity were quantified using a NanoDrop spectrometer (Thermo Fisher Scientific, Waltham, MA, USA). Only isolated RNA with a 260/280 ratio between 1.9 and 2.1 was used.

### 2.3. RNA Library Production and Sequencing

RNA concentration was measured using a Qubit 3.0 with the RNA BR Assay Kit (Thermo Fisher Scientific, Waltham, MA, USA). RNA quality was assessed using the Agilent 2100 Bioanalyzer (Agilent Technologies, Santa Clara, CA, USA), and only samples with RIN > 8 were used for RNA sequencing. Total RNA-seq NGS libraries were prepared using the NEBNext^®^ Ultra II Directional RNA Library Prep Kit (New England Biolabs, Ipswich, MA, USA) according to the manufacturer’s protocol, without further modifications. rRNA molecules were removed from total RNA using NEBNext^®^ rRNA Depletion Kit v2 (Human/Mouse/Rat). Briefly, 10 ng of total RNA was used as input for initial hybridization to rRNA probes, followed by RNase H and DNase I digestion steps, and a final cleanup. Enriched non-rRNA species were used for cDNA first- and second-strand synthesis, followed by purification on AMPure XP beads (Beckman Colter, Brea, CA, USA) to separate blunt-ended dsDNA from unincorporated nucleotides. cDNA was then adenylated at its 3′ ends, followed by ligation of indexing adapters specific for each sample. The DNA fragments were enriched with adapter molecules on both ends using 12 cycles of PCR, followed by purification on AMPure XP beads. The library size and quality were assessed on High Sensitivity DNA Bioanalyzer chips (Agilent). Next, NGS libraries were quantified using the KAPA Library Quantification Kit and normalized to 4 nM. Libraries were pooled and denatured in freshly prepared 0.2 N NaOH, then diluted with HT1 buffer to a final concentration of 1.8 pM. As a sequencing control, the library pool was spiked with 1% (*v*/*v*) denatured PhiX library at 1.8 pM and loaded onto the reagent cartridge. The sequencing run was performed on a NextSeq 500 (Illumina, San Diego, CA, USA) in paired-end mode with a read length of 2 × 76 bp. Illumina BaseSpace-created FASTQ files were used for further analysis.

### 2.4. Gene Analysis

Raw sequence data (FASTQ) were first assessed for quality using FastQC (v0.11.9) [20]. Reads were then processed using Trimmomatic (v0.39) [21] to remove adapter sequences; low-quality bases from both 5′ and 3′ ends were trimmed (Phred quality score < 30), and reads with a length <20 bp and a mean Phred quality score < 30 were discarded (Trimmomatic v0.39). The remaining high-quality reads were aligned to the Homo sapiens GRCh38.p13 reference genome using HISAT2 (v2.2.2) [22], following genome indexing with hisat2-build. Alignment files were converted (SAM) to BAM, then sorted and indexed using SAMtools (v1.7) [23]. Gene-level quantification was performed using HTSeq [24] (htseq-count) to generate raw read counts. Initially, 60,566 gene features were detected; after filtering out low-count transcripts, 52,369 gene features were retained for downstream analysis. Differential expression analysis was performed using the DESeq2 (v.1.34) [25] package in R. Raw counts were used because DESeq2 requires integer-count data and performs internal normalization using the median-of-ratios method to account for differences in library size and RNA composition. Differentially expressed genes (DEGs) between opiate-exposed placenta and control placenta were identified using a threshold of a Benjamini–Hochberg-adjusted *p*-value < 0.05, with a log2 fold change > 1.0 to focus on important genes of interest and a cutoff of >0.7. Visualization of differential expression was performed using the ggVolcanoR application. Functional interpretation, including canonical pathway enrichment and network analysis, was performed using Ingenuity Pathway Analysis (IPA) (v22.0), in which pathway directionality (z-scores) was inferred from effector-gene expression patterns and significance determined using right-tailed Fisher’s exact test (*p* ≤ 0.05), with predicted activation/inhibition determined by z-score algorithms (z ≥ 2.0).

## 3. Results

### 3.1. Patient Data Indicate the Placentas Are Demographically Aligned Across Key Baseline Parameters

To understand the effect of opioid exposure on the human placenta in Hawai’i’s unique and diverse population, placentas were selected that had been either exposed or not exposed to opioids. These had previously been collected and stored with medical records in the Hawai’i Reproductive Biospecimen Repository. This biorepository holds 9000 matched maternal blood, fetal blood, and placenta samples, collected following delivery at Kapi’olani Medical Center for Women and Children in Honolulu, Hawai’i. A smaller, more precise selection strategy was used, rather than a larger, less well-defined patient cohort, i.e., deep and narrow versus wide and shallow (Table 1). As is typical of mothers who take opioids during pregnancy, information regarding the amount and type of opioid taken during the pregnancy was self-reported, minimally provided, and therefore limited in the associated medical records.

However, there were no statistical differences between the two groups of women from whom the placentas were collected—opioid-exposed or non-opioid-exposed—for maternal age, BMI, gestational age at delivery, mode of delivery, sex of baby, weight of baby, parity, or ethnicity. Sixty-seven percent of the patients in the opioid-exposed group delivered vaginally, whereas 66.7% of the control patients delivered by Cesarean section. The mothers were of similar ethnicity, reflecting the diverse population of Hawai’i, although one mother in the opioid-exposed group was Caucasian. There was one smoker in each group. The opioid group had one mother who had used marijuana, and within the opioid-exposed group, documented exposures included cases of prescribed opioid-maintenance therapies as well as illicit opioid use. Although 50% of the opioid group had preterm labor, 33.3% of the control group also did; this led to there being no statistical difference in gestational age at delivery or neonatal weight between the groups (Table 1).

### 3.2. RNAseq Predicts Gene Changes Reflective of a Decrease in Cellular Metabolism in Opioid-Exposed Placenta

**Figure 1:**
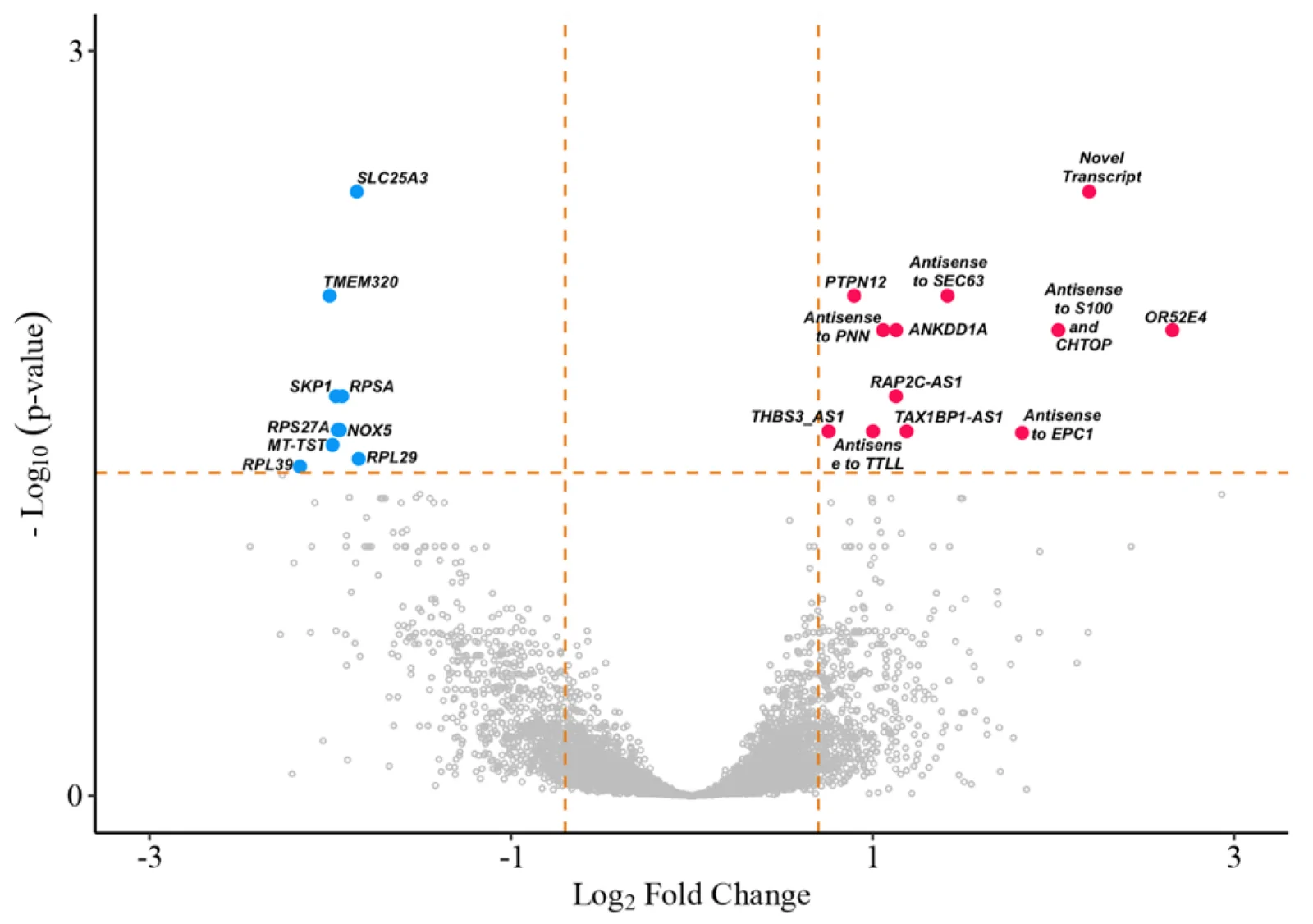
Volcano plot of the top 2500 differentially expressed genes (DEGs) in opioid-exposed placenta compared to non-exposed placenta. The y-axis represents the statistical significance (−Log10 *p*adj value), and the x-axis represents the magnitude of change (Log2 Fold Change). Significantly differentially expressed genes (DEGs) were identified using an adjusted *p*-value (*p*adj) < 0.05. An absolute log2 fold-change threshold of >1.0 was used to identify important significant genes. In the volcano plot, genes meeting these criteria are color-coded to indicate directionality: red denotes upregulated genes and blue denotes downregulated genes. Genes that did not meet both the significance and magnitude thresholds are represented in gray (*n* = 6 in each group). A total of 10,697 features are displayed out of 52,368 detected, representing 20.43% of the data.

Following RNAseq, 52,369 gene features were detected and retained after low-count filtering from the comprehensive RNA library (full list available in Supplemental Data Table S1). The top 20% of differentially regulated genes are depicted in Figure 1 to show the distribution of the most potentially interesting genes in the log2 fold change vs. −log10 of the *p*-value. Differential Expression Sequencing 2 (DESeq2) analysis identified 21 significantly (adjusted *p*-value < 0.05) changed genes that were considered high-confidence candidates. Twelve of these statistically significant genes showed increased expression with opioid exposure during the pregnancy, and nine were associated with decreased expression in the opioid-exposed placenta compared to non-opioid exposure (Table 2 and Figure 1). Interestingly, five of the significantly upregulated genes were antisense transcripts: *SEC6* Protein Translocation Regulator 3 (*SEC63*); *S100* Calcium-Binding Protein A1 (*S100A1*); *Pinn*, Desmosome-Associated Protein (*PNN*); and Enhancer of Polycomb 1 (*EPC1*). The most significantly upregulated gene was a ‘novel transcript’, followed by Protein Tyrosine Phosphate Non-Receptor Type *12* (*PTPN12*), Olfactory Receptor Family 52 Subfamily E Member 4 (*OR52E4*), and Ankyrin Repeat and Death Domain-Containing 1A (*ANKDD1A*). These were closely followed by the upregulation of *RAP2C* Antisense RNA 1 (*RAP2C-AS1*), *Tax1*-Binding Protein 1 Antisense RNA 1 (*TAX1BP1-AS1*), and Thrombospondin 3 Antisense RNA 1 (*THBS3-AS1*). The most significantly downregulated genes were Solute Carrier Family 25 Member 3 (*SLC25A3*) and Transmembrane Protein 230 (*TMEM230*), as well as Ribosomal Protein SA (*RPSA*), Ribosomal Protein 29 (*RPL29*), and Ribosomal Protein 39 (*RPL39*). Other key genes that were significantly decreased include S-Phase Kinase-Associated Protein 1 (*SKP1*), NADPH Oxidase 5 (*NOX5*), and Mitochondrially Encoded tRNA-Ser (*UNC*) *1* (*MT-TS1*) (Table 2).

### 3.3. Opioid-Exposed Placentas Show Low Genetic Variation for Top Fold-Change Genes

**Figure 2:**
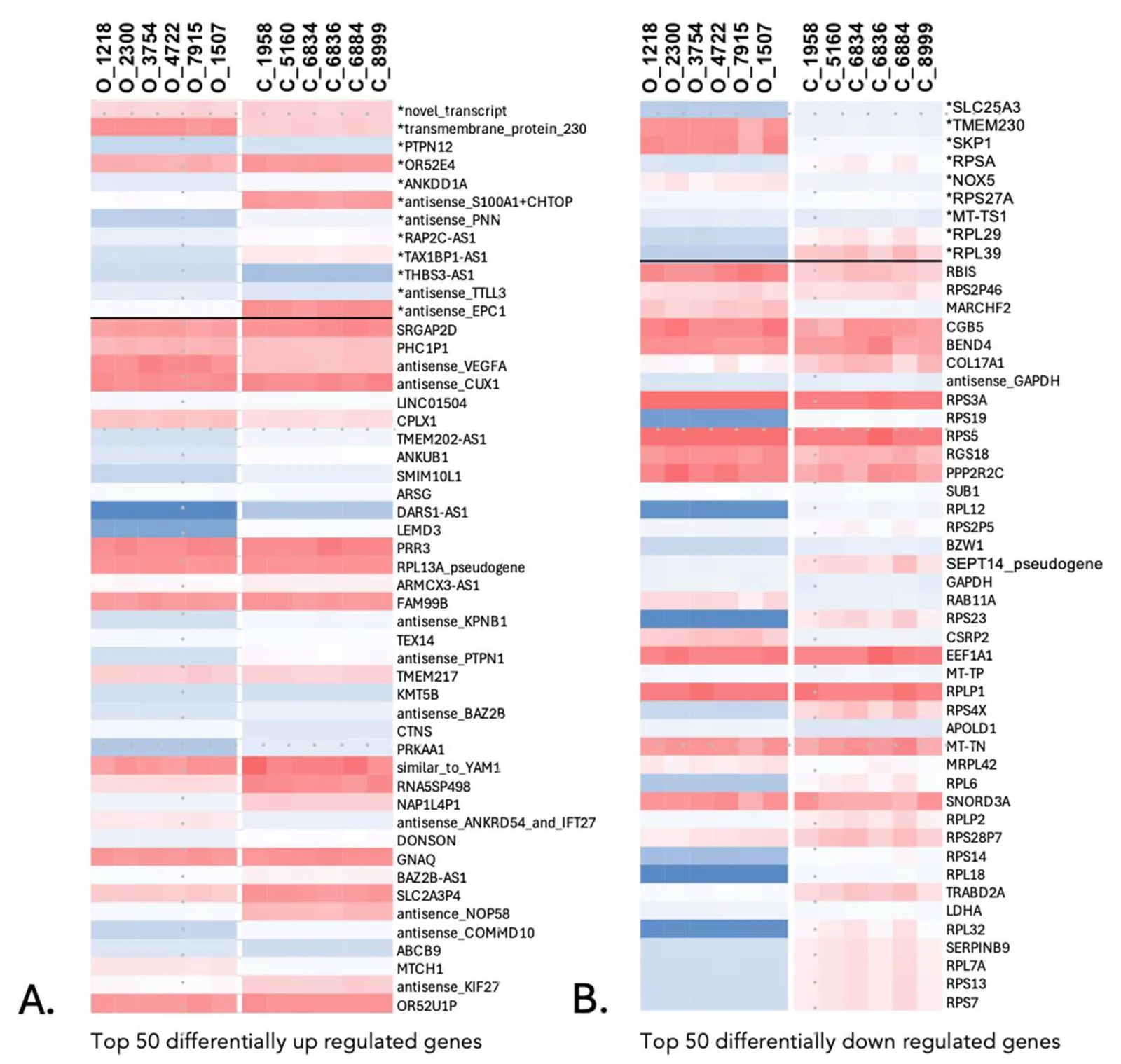
Transcriptomic profiling predicts intra-group homogeneity of gene expression. Opioid-exposed (O) and non-exposed (C) patient populations (*n* = 6 in each group). (**A**) Heat map representing the top 50 upregulated genes and (**B**) the top 50 downregulated genes (DEGs) based on [DESeq2/EdgeR] analysis. Data are presented as calculated Z-scores [log2-transformed] for each patient, relative to the group average for that gene in the raw-normalized (individual-gene) data. Z-scores are presented to highlight expression consistency across the two study groups (*n* = 6 in each group), despite potential heterogeneity in the opioid-exposed group. Blue represents a negative number, and red represents a positive number compared to the group average. The darker color indicates increased deviation. * Denotes significantly changed genes compared to non-opioid exposure.

Because of the focused sampling approach, the data quality and probe transcriptomic alignment across patient samples within each group were assessed further; the data were log-transformed, and z-scores were calculated. This allowed for the determination of each patient’s degree of change relative to the group average. This gave a numerical measure of each patient’s deviation from the group, allowing for assessment of how closely patients within each group were demographically aligned on key baseline parameters. This was particularly important given the sparse clinical data regarding the dosing of opioid exposure, and the potential for heterogeneity that often exists in self-reported opioid use samples. Each patient’s data were plotted in each group as heat maps of the top 50 upregulated and downregulated genes to visualize transcriptomic alignment within each group (Figure 2). These data show that patient-to-patient variation within both groups was low (for the top 100 genes), as hierarchical clustering demonstrated relative transcriptomic alignment within groups. Interestingly, these top 100 genes showed greater alignment in the opioid-exposed group (A and B, left side) than in the control group (A and B, right side), as indicated by the more uniform color across the gene row for that group of six patients (Figure 2). A heat map for the expression level of the top 100 genes for each gene transcript was also produced (Supplemental Figure S1).

### 3.4. Ingenuity Pathway Analysis Indicates Opioid Exposure Pushes Cells into Survival Mode

**Figure 3:**
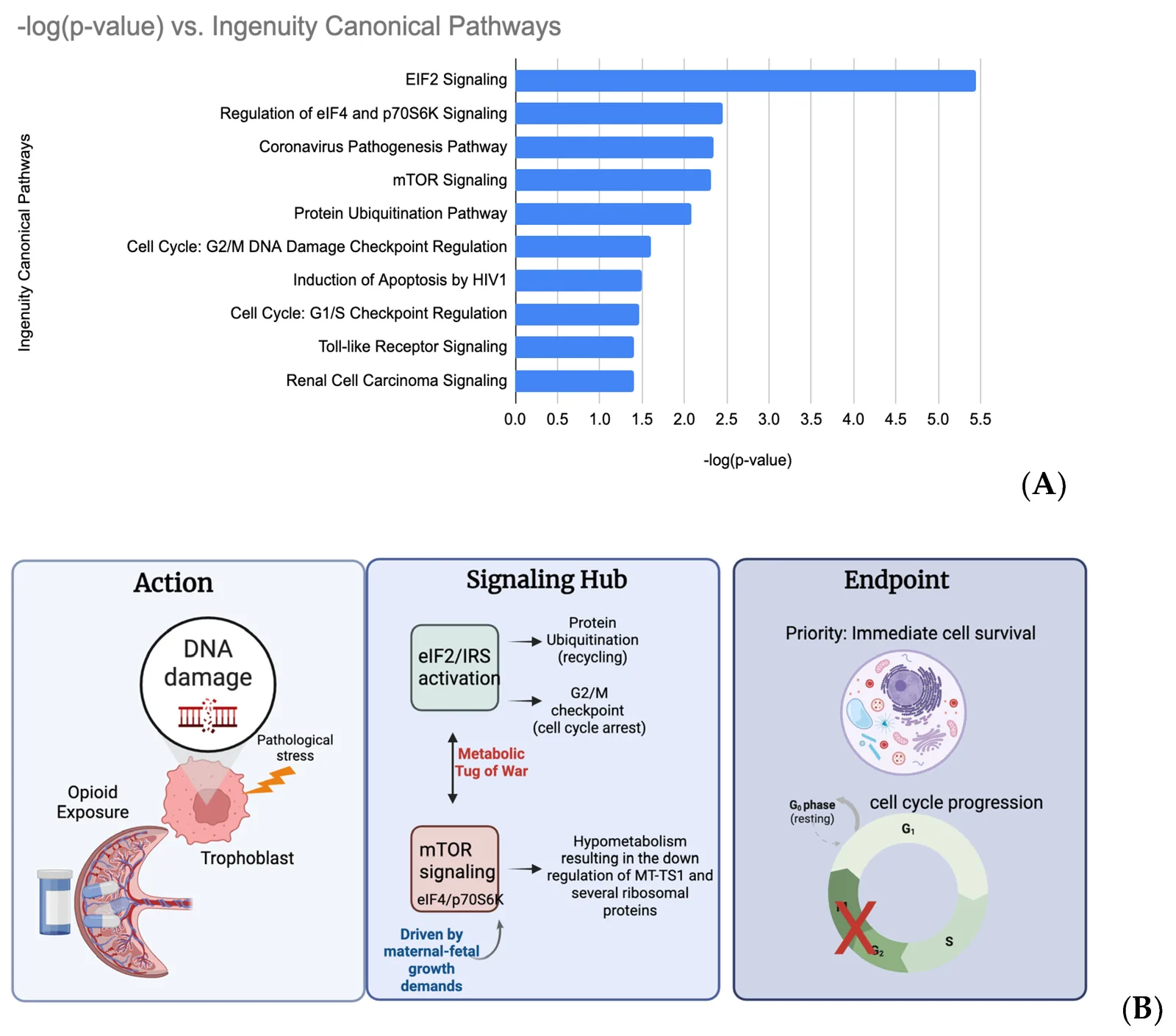
Canonical pathway prediction of differentially expressed genes in opioid-exposed placenta. (**A**) Ingenuity Pathway Analysis (IPA) modeling of systemic metabolic pathways. This approach predicted 10 significantly enriched canonical pathways for consideration. The Eukaryotic Translation Initiation Factor-2 (EIF2) signaling pathway was the most significantly enriched, followed by regulation of Eukaryotic Translation Initiation Factor 4/p70 Ribosomal Protein S6 Kinase 1 (eIF4/p70S6K) and Mechanistic Target of Rapamycin Kinase (mTOR) signaling. Pathological enrichment in cell-cycle checkpoints (G2/M and G1/S) and Toll-like receptor signaling was also inferred. (**B**) Proposed hypothesis of predicted regulatory states based on IPA predictions. Graphical representation of canonical pathways and biological functions predicted to be modulated by opioid exposure in the placenta. Analysis reveals positive Z-scores (predicted activation) for stress-sensing mechanisms, including EIF2, mTOR, and eIF4/p70S6K signaling, as well as the protein ubiquitination pathway and the G2/M DNA damage checkpoint. Conversely, negative Z-scores suggest a downregulation of mitochondrial transcriptional activity and ribosomal systems. These integrated pathway shifts are consistent with a coordinated cellular stress response (Created in BioRender. Wright, C. (2026) https://BioRender.com/zqi629m).

After identifying several significantly differentially expressed genes and observing consistent expression across placentas within the experimental groups, pathway analysis was conducted using Ingenuity Pathway Analysis (IPA) on all RNA-seq data from the library. The statistically significant canonical pathways that were identified in the opioid-exposed placentas compared to the non-opioid-exposed placentas were noted. This analysis was performed with a cutoff of log (*p*-value) ≥ 1.3. Ten pathways of interest were identified (Figure 3A). The most significant pathway inferred was Eukaryotic Translation Initiation Factor 2 (EIF2) signaling, with a log *p*-value of −5.5. This pathway, along with four others, was functionally clustered within the biological realm of cellular metabolism. These included Mechanistic Target of Rapamycin Kinase (mTOR) signaling, Eukaryotic Translation Initiation Factor 4/p70 Ribosomal Protein S6 Kinase 1 (eIF4/p70S6K) regulation, ubiquitination, and the G2/M checkpoint. Other predicted pathways included coronavirus pathogenesis, HIV-induced apoptosis, Toll-like receptor signaling, and renal cell carcinoma signaling (Figure 3A). Enriched IPA canonical pathways such as coronavirus pathogenesis or renal cell carcinoma reflect shared underlying molecular machinery (e.g., ribosomal protein suites, heat-shock response, and cell-cycle checkpoints) and do not imply active viral infection or oncogenesis in placental tissue. The combination of significantly inferred upregulated and downregulated pathways helped to generate a behavior prediction model of placental cells in response to opioid exposure using the gene expression data. The bioinformatic pathway prediction suggested that the cells may be entering a survival-under-stress mode (Figure 3B).

### 3.5. Network Analysis Points to Two Complementary Inhibited Networks, Suggesting Translational and Metabolic Stasis

**Figure 4:**
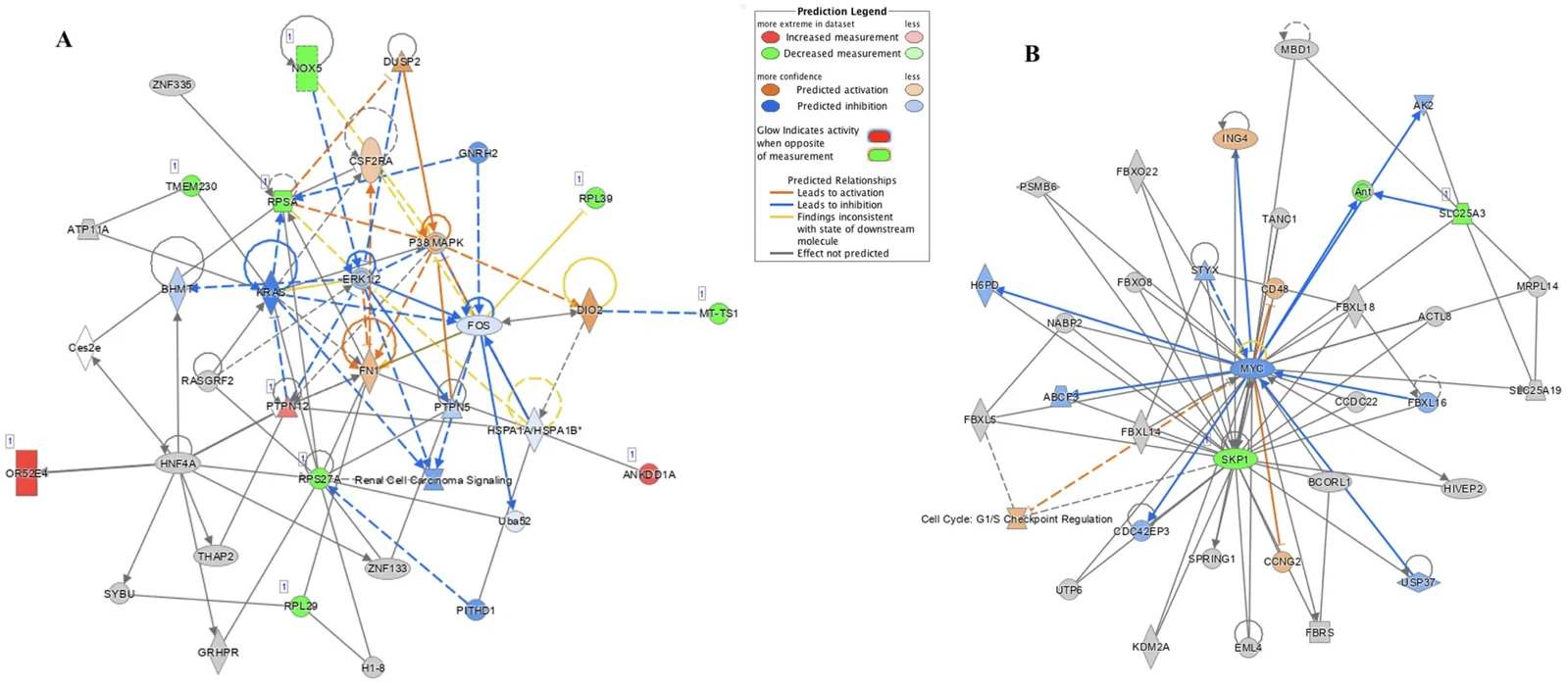
Biological interactions with seed molecules and connections based on significant differentially expressed genes and canonical pathway analysis. Each connection represents known relationships between the molecules, found in the Ingenuity Knowledge Base. Two distinct networks were identified: (**A**) Cancer, Gastrointestinal Disease, Organismal Injury and Abnormalities (MAP Kinase ERK1/2 Hub), and (**B**) Cancer, Gene Expression, Organismal Injury and Abnormalities, Reproductive System Disease (MYC Hub).

To complete the analysis, Ingenuity Network Analysis (INA) was performed. This enabled identification of two major network hubs, which in turn supported further development of the predictive model of opioid effects on the human placenta. The two networks predicted by the Ingenuity Knowledge Base were (1) Cancer, Gastrointestinal Disease, Organismal Injury and Abnormalities, and (2) Cancer, Gene Expression, Organismal Injury and Abnormalities, Reproductive System Disease (Figure 4). The first predicted network illustrates a central hub of Mitogen-Activated Protein (MAP) Kinase signaling driven by Extracellular Signal-Related Kinase 1/2 (ERK1/2), Kirsten Rat Sarcoma Virus Proto-Oncogene GTPase (KRAS), and Fos Proto-Oncogene, AP-1 Transcription Factor subunit (FOS) (Figure 4A). The INA supports this network, as it is surrounded by statistically significant hit genes from the gene analysis: *MT-TS1*, *RPL39*, *RPSA*, *TMEM230*, and *NOX5*. This INA analysis also predicted that this network would be inhibited. The second predicted network was also thought to be inhibited. However, the central hub of this network was MYC (Figure 4B), which was linked in proximity to other significant genes of interest, including *SKP1*, *SLC25A3*, and Adenine Nucleotide Translocator.

## 4. Discussion

While opioid exposure during pregnancy continues to be a critical issue in the US [1], the understanding of how opioids may reprogram the placenta remains incomplete, complicating efforts to mitigate the effects of these drugs. The transcriptomics data from opioid-exposed placentas from Hawai’i collectively suggest that these placental cells may experience cellular stress and could respond by entering a putative cellular survival mode (Figure 3B).

While this study provides a high-resolution vantage point on the human placental response to maternal opioid use, several structural and biological limitations inherent to human translational discovery must be acknowledged. First, the primary clinical constraints of this study are the relatively small cohort size and the limited granular characterization of the participants’ specific opioid exposure. Because these clinical specimens are completely de-identified to protect participant privacy under institutional IRB frameworks, retroactive medical record extraction to detail daily dosages, specific drug compounds, or precise timelines remains procedurally bounded. Consequently, subgroup stratification across specific opioid modalities (e.g., methadone vs. buprenorphine vs. illicit formulations) was statistically underpowered in this discovery cohort (*n* = 6 per group). Nevertheless, the emergence of a robust, statistically conservative (*FDRq* < 0.05) core pathway signature raises the hypothesis that these altered networks represent a conserved, convergent placental response to opioid-class exposure rather than an artifact of a single drug formulation.

To partially mitigate potential confounders inherent in larger, more heterogeneous clinical data, the patient groups were intentionally selected to be demographically aligned across key baseline parameters. There was no statistical difference between the opioid-exposed and non-opioid-exposed groups across typical clinical metadata (Table 1), allowing this discovery-phase cohort to yield a clean, high-resolution biological signal.

Given the biological covariates, the sample size of this discovery cohort precluded a robust, sex-stratified analysis, despite fetal sex being a well-established driver of placental dimorphism. To maintain statistical rigor and avoid underpowered subgroup inferences, fetal sex was not treated as a covariate in the analytical pipeline. Crucially, despite the real-world heterogeneity typical of untracked gestational exposures, the interpatient variability analysis showed that the opioid-exposed group exhibited strong alignment across the top significantly differentially expressed genes (Figure 2A,B). The emergence of highly interconnected transcriptomic hubs supports the hypothesis that maternal opioid exposure, rather than individual clinical variability, was a major contributing factor to the observed changes in gene expression, representing a putative adaptive molecular phenotype of placental stress. Furthermore, a standardized placenta-processing protocol helped minimize potential confounding from handling artifacts, supporting the interpretation that differences in mitochondrial gene transcripts in the comprehensive RNA-seq library more closely reflect the underlying biological state than ex vivo cellular stress randomly induced during placenta collection.

The lack of orthogonal bench validation (e.g., RT-qPCR, Western blot, or IHC) and the potential for unmeasured confounding from co-occurring exposures (such as tobacco or secondary substances) as inherent boundaries of this discovery-phase study should also be acknowledged. While the analytical pipeline was statistically optimized to prioritize high-confidence transcriptomic networks, these bioinformatic projections represent hypothesized pathways rather than confirmed functional mechanisms. Consequently, prospective, longitudinal replication in larger, multi-site cohorts will be necessary to fully validate these regulatory hubs and establish their precise translational utility.

The data analysis identified several statistically significant differentially expressed genes, with high overall expression alignment across the opioid-exposed placenta group. Among these, *PTPN12* emerged as the second most significantly upregulated transcript. Recognized as a core molecular brake on cellular signaling, *PTPN12* encodes a protein tyrosine phosphatase that regulates cell migration by inhibiting focal adhesion dynamics [26]. In the context of placental biology, this observed upregulation raises the hypothesis of impaired trophoblast invasion, which could represent an underlying transcriptomic contributor to the placental insufficiency frequently observed in opioid-exposed pregnancies [27]. Furthermore, given its established role in dampening key receptor tyrosine kinase pathways (such as IGF and EGF signaling) [28], elevated *PTPN12* expression may reflect an attenuated capacity of the fetal-placental unit to respond to baseline developmental growth signals. Intriguingly, beyond its placental functions, *PTPN12* has also been implicated in neural development and synaptic plasticity [29], highlighting a potential gene of interest for future cross-tissue studies along the placenta–brain axis [30].

Several antisense transcripts were also statistically significantly upregulated in the opioid-exposed cohort, raising the possibility of targeted endogenous biological repression. Of particular interest, upregulation of the antisense transcript complementary to *SEC63*, a key component of the endoplasmic reticulum (ER) protein translocation machinery, was observed. Hypothetically, antisense-mediated modulation of *SEC63* transcript stability could contribute to downstream ER stress, a pathway frequently associated with opioid-associated cellular strain and placental dysfunction [19,31,32]. This transcriptomic signal aligns with the observed concurrent downregulation of structural ribosomal subunits (*RPL*/*RPS*) and mitochondrial solute carriers (e.g., *SLC25A3*). Collectively, these integrated molecular shifts support a model of coordinated attenuation across cellular translational and bioenergetic machinery. Such antisense transcripts are hypothesized to function as regulatory fine-tuners, analogous to mechanisms reported in acute inflammatory cascades [33], potentially stabilizing target pathways in a repressed state. While elevated antisense expression co-occurs with the downregulation of corresponding sense transcripts and translational pathways in the dataset, future functional assays, including RNA–RNA interaction studies and targeted knockdown experiments in primary trophoblast models, will be essential to demonstrate true post-transcriptional silencing mechanisms.

Beyond individual gene perturbations, the canonical pathway analysis (via IPA) yielded a bioinformatic model depicting potential coordinate shifts in the placental transcriptomic response to opioid exposure (Figure 3). Interestingly, the dataset predicted parallel enrichment signatures across both the EIF2 and mTOR pathways. This dual-pathway signature suggests the cell may attempt to maintain anabolic growth (via mTOR) while simultaneously managing an emerging stress profile (via EIF2). Such a signature could reflect a cellular state of uncoupled metabolic demand and translational arrest [34]. Under sustained opioid-induced stress, the data suggest the hypothesis that mTOR signaling may remain active in a compensatory effort to sustain basal cellular bioenergetics and survival [35], while the parallel eIF2-mediated Integrated Stress Response (ISR) could act as an upstream structural brake on heavy ribosomal translation to conserve energy and prevent proteotoxic stress [36,37]. This transcriptomic profile aligns with a state of compensatory hypermetabolism, a phenomenon frequently documented in cases of Fetal Growth Restriction [38], in which the placenta operates at its maximum physiological capacity to compensate for environmental stressors. Furthermore, the identification of the eIF4 and p70S6K signaling pathways, critical downstream effectors of protein synthesis, aligns with a proposed model in which the predicted activation of EIF2 serves as a candidate regulatory stop signal [36]. In light of this hypothesized metabolic strain, the predicted activation signature of the ubiquitination pathway is noteworthy and may reflect an increased cellular requirement to clear misfolded or damaged proteins [39]. Finally, the predicted activation of the cell-cycle G2/M damage checkpoint pathway raises the possibility of a programmed pause in replication, hypothetically engaged to assess or mitigate genotoxic stress [40]. When viewed in concert, these bioinformatics predictions point to a hypothetical, coordinated transcriptomic architecture in which cellular resources appear to be prioritized toward survival and repair mechanisms over anabolic processes [41,42,43]. However, because these bioinformatic algorithms are inherently modeling tools guided by transcriptomic directionality and curated literature repositories, these findings should be interpreted as statistically integrated hypotheses. Nevertheless, they provide a standardized framework for meta-analytical harmonization across cohorts, offering a robust, hypothesis-generating baseline to guide future functional bench validation.

Informed by the IPA computational analysis, two candidate biological networks were modeled (Figure 4) depicting computational projections of coordinated transcriptomic suppression of placental growth pathways following opioid exposure. Rather than redundantly visualizing differential expression, this network analysis provides a hypothesis-generating framework by modeling functional hubs and regulatory bottlenecks that may be underrepresented in a linear gene list. By mapping these interdependencies, it is possible to move beyond individual gene perturbations to highlight putative systems-level alterations that may characterize the placental phenotype. The first network (Figure 4A), statistically predicted by the model, is characterized by a chronic stress-signaling axis (ERK1/2, p38 MAPK) associated with candidate pathways of translational attenuation. This signaling cascade parallels the downregulation of structural ribosomal machinery and reactive oxygen species-buffering genes (*NOX5* and *TMEM230*), as well as the predicted inhibition of *KRAS* and *FOS*. Combined with the hypothetical role of *PTPN12* as a molecular brake on growth signaling, this depressed baseline ERK/MAPK signature is consistent with established models of impaired trophoblast proliferation, blunted syncytial differentiation, and compromised nutrient transport capacity [44,45]. Together, these transcriptomic alterations support a prospective model of integrated cellular strain, offering a plausible bioinformatic baseline for the cellular quiescence and placental stasis frequently noted in opioid-exposed pregnancies [46,47]. While the high-throughput sequencing prioritizes these core transcriptional hubs as high-value candidates, future functional proteomics and targeted phosphoproteomic studies will be required to validate the exact post-translational phosphorylation states of the mTOR/EIF2 networks. This network approach enriches the dataset with critical layers of biological context; while differential expression identifies individual candidate components of the opioid response, network analysis models a predicted regulatory architecture through which these components are hypothesized to interact. This allows for the prioritization of convergent hubs, such as *MYC* and *PTPN12*, that may represent high-leverage regulatory nodes regulating placental adaptation under stress.

**Figure 5:**
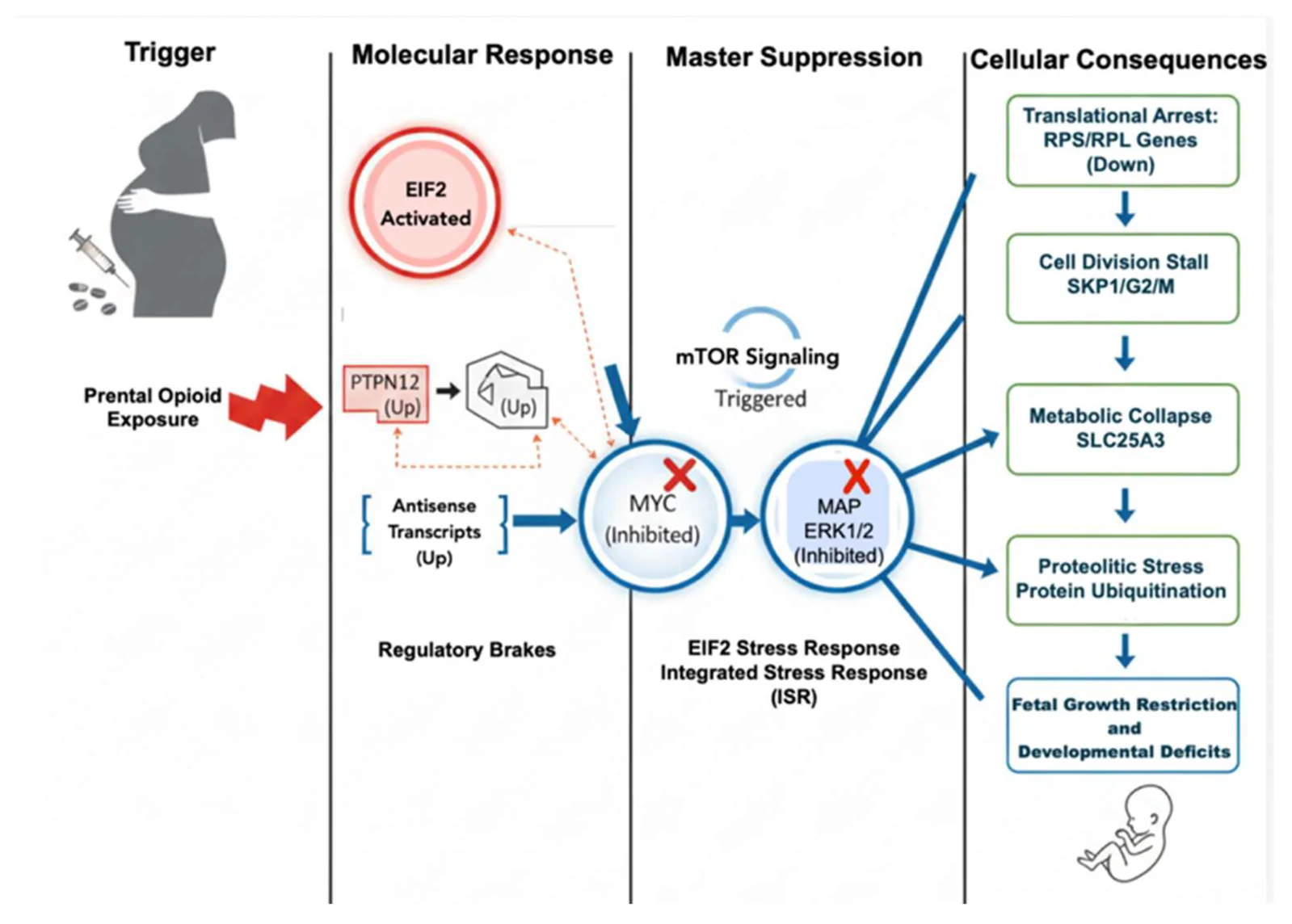
Suggested model of a potential mechanism of opioid-induced placental dysfunction via the Integrated Stress Response (ISR). Schematic representation of the molecular cascades triggered by prenatal opioid exposure in the placenta, as suggested by the data. (**Left**) Trigger: Prenatal opioid exposure initiates a multifaceted stress response at the maternal–fetal interface. (**Center-Left**) Opioid exposure may induce the upregulation of Protein Tyrosine Phosphate Non-Receptor Type 12 (*PTPN12*) and antisense transcripts, and activate Eukaryotic Translation Initiation Factor 2 (EIF2) pathways. The concurrent predicted increase in antisense transcripts could act as a regulatory brake, thereby inhibiting the Myelocytomatosis Proto-Oncogene (MYC) signaling hub. (**Center-Right**) eIF2 signaling could then be triggered, with the ISR serving as a master suppression mechanism that inhibits central regulatory hubs. (**Right**) This integrated molecular suppression may then result in cellular deficits, including translational arrest (supported by the downregulation of *Ribosomal protein* (*RPS*/*RPL*) genes), cell-cycle arrest (associated with S-Phase Kinase Associate Protein 1/G2/M (*SKP1*)), metabolic consequences (Solute Carrier Family 25 Member 3 (*SLC25A3*)), and proteolytic stress (increased protein ubiquitination). Collectively, this model is based on the transcriptomics data, which suggests that these placental changes may provide a novel mechanistic basis for some of the observed fetal growth restriction and developmental deficits associated with prenatal opioid exposure. Figure structure drawn using Nano Banana Pro under instruction by CKW and annotated by CKW using Preview.

Complementing this, the second predicted network (Figure 4B) delineates a bioinformatic model of metabolic and cell-cycle constraints centered on the predicted inhibition of *MYC*, a master regulator of cellular proliferation [48]. The simultaneous downregulation of *SLC25A3* and *SKP1* [49] aligns with a hypothesized concurrent reduction in mitochondrial energy production and Skp1-Cullin1-F-box protein complex-mediated cell-cycle progression [50]. Furthermore, the predicted inhibition of Hexose-6-Phosphate Dehydrogenase (*H6PD*) raises the possibility of an altered shift in glucose metabolism [51,52]. Ultimately, these two networks support the hypothesis that gestational opioid exposure may induce a transcriptomic state of metabolic and translational strain, providing a putative molecular framework for the systemic restriction of fetal growth (Figure 5).

Computational modeling is a valuable complementary tool for generating prioritized hypotheses before downstream functional validation. In a system as complex as the human placenta, bioinformatic network mapping provides a data-driven framework to prioritize high-leverage nodes (such as *MYC* or *PTPN12*) for targeted empirical interrogation. The working model (Figure 5) offers a proposed, systems-level architecture depicting candidate regulatory shifts associated with gestational opioid exposure. While this model requires prospective refinement, it provides a unifying scaffold on which future empirical data can be systematically organized and evaluated via targeted bench validation. Moving forward, cell-type deconvolution, single-cell RNA sequencing, and spatial transcriptomics represent priority strategies to resolve cell-type-specific responses, building upon this bulk transcriptomic baseline. Furthermore, integrating targeted proteomic or metabolomic profiling in future cohorts will be instrumental in functionally validating the predicted downstream readouts of the mapped eIF2\α/ISR and mTOR signaling axes.

## 5. Conclusions

In summary, the transcriptomic profiling provides a localized model of coordinated molecular shifts in the human placenta following prenatal opioid exposure within a real-world clinical cohort, characterized by putative pathways of translational attenuation and metabolic strain. Network analysis delineates a predicted stress-signaling architecture centered on suppression of p38 MAPK/ERK1/2 signaling, mirroring the significant downregulation of key ribosomal structural subunits (*RPS27A*, *RPL29*, and *RPL39*) and upstream regulatory hubs. Collectively, these findings suggest a foundational transcriptomic landscape that indicates gestational opioid exposure may disrupt core biosynthetic pathways supporting placental function. Clinically, characterizing these candidate regulatory nodes is an essential step toward identifying high-priority molecular biomarkers of placental stress, which may ultimately aid in risk stratification of vulnerable pregnancies and guide future targeted mechanistic studies to improve maternal–fetal outcomes.

## Figures and Tables

**Table 1:** Patient demographic data for opioid-exposed and non-opioid-exposed placenta samples from the Hawai’i Biospecimen Repository (*n* = 6 in each group).

	Non-Opioid-Exposed	Opioid-Exposed	Significance *p* < 0.05 Fisher’s Exact Test
Maternal age (years)—average (SD)	26.6 (6.12)	22.6 (4.16)	0.233
Parity (number of children)—average (SD)	2.5 (1.97)	1 (1.095)	0.135
Maternal BMI (kg/m^2^)—average (SD)	23.26 (2.7)	23.26 (1.42)	0.999
Gestation age at delivery (weeks)	35.67 (5.64)	37.67 (3.01)	0.462
Mode of delivery—vaginal (%)/C-section (%)	33.3/66.7	66.7/33.3	-
Sex of baby—% male/% female	64/36	50/50	-
Baby weight (grams)—average (SD)	2792.6 (1220.7)	2689.83 (644.9)	0.859

**Table 2:** The top 21 significantly differentially expressed genes (DEGs) in opioid-exposed human placentas compared to non-opioid-exposed placentas. These are also depicted as blue (down) and red (up) in Figure 1 (*n* = 6 in each group).

Gene Name	Log2 Fold Change	*p*adj Value	Predicted Change
Novel Transcript	2.1979	0.00368	Up
Antisense to *SEC63*	1.4141	0.00967	Up
*PTPN12*	0.8979	0.00967	Up
*OR52E4*	2.6583	0.01331	Up
*ANKDD1A*	1.301	0.01331	Up
Antisense to *S100A* and *CHTOP*	2.0271	0.01331	Up
Antisense to *PNN*	1.0585	0.01331	Up
*RAP2C-AS1*	1.1293	0.02456	Up
*TAX1BP1-AS1*	1.1879	0.03401	Up
*THBS3_AS1*	0.7564	0.03402	Up
Antisense to *TTLL3*	1.0023	0.02456	Up
Antisense to *EPC1*	1.827	0.03449	Up
*SLC25A3*	−1.8529	0.00368	Down
*TEM230*	−2.0041	0.00967	Down
*SKP1*	−1.9683	0.02456	Down
*RPSA*	−1.9342	0.02456	Down
*NOX5*	−1.9461	0.03362	Down
*RPS27A*	−1.9588	0.03362	Down
*MT-TS1*	−1.9876	0.03858	Down
*RPL29*	−1.8427	0.04395	Down
*RPL39*	−2.1677	0.04712	Down

## Data Availability

All data are freely available in the Supplementary Materials.

## Supplementary Materials

The following supporting information can be downloaded at: https://www.mdpi.com/article/10.3390/cimb48090900/s1.

## Abbreviations

The following abbreviations are used in this manuscript:

AFP	Alpha Fetoprotein
ANKDD1A	Ankyrin Repeat and Death Domain-Containing 1A
Ant	Adenine Nucleotide Translocator
APOB	Apolipoprotein B
APOE	Apolipoprotein E
ATP6V0A4	ATPase H+ Transporting V0 Subunit A4
CCDC12	Coiled-Coil Domain-Containing 120
CEACAM	Carcinoembryonic Antigen-related Cell Adhesion Molecules
CYP1A1	Cytochrome P450, Family 1, Subfamily A, Polypeptide 1
DEGs	Differentially Expressed Genes
DESeq2	Differential Expression Sequencing 2
eIF2	Eukaryotic Translation Initiation Factor 2
eIF4	Eukaryotic Translation Initiation Factor 4
EPC1	Enhancer of Polycomb 1
ERK1/2	Extracellular Signal-Related Kinase 1/2
FAM20	Family With Sequence Similarity 20, Member C
FGR	Fetal Growth Restriction
FOS	Fos Proto-Oncogene, AP-1 Transcription Factor Subunit
GCM1	Glial Cells Missing Transcription Factor 1
GRCh38	Genome Reference Consortium Human Build 38
GRIN2A	Glutamate [NMDA] Receptor Subunit Epsilon-1
H2A	Histone H2A
H6PD	Hexose-6-Phosphate Dehydrogenase/Glucose 1-Dehydrogenase
HBB	Hemoglobin Subunit Beta
HISAT2	Hierarchical Indexing for Spliced Alignment of Transcripts
HTSeq	High-Throughput Sequencing
HUNK	Hormonally Upregulated Neu-Associated Kinase
IL1RL1	Interleukin 1 receptor-like 1
INA	Ingenuity Network Analysis
INBRE	IDeA Networks of Biomedical Research Excellence
IPA	Ingenuity Pathway Analysis
ISR	Integrated Stress Response
KRAS	Kirsten Rat Sarcoma Virus Proto-Oncogene GTPase
MAP	Mitogen-Activated Protein
MT-TS1	Mitochondrially Encoded TRNA-Ser (UCN) 1
mTOR	Mechanistic Target of Rapamycin Kinase
MYC	Myelocytomatosis Proto-Oncogene, BHLH Transcription Factor
NOWS	Neonatal Opioid Syndrome
NOX5	NADPH Oxidase 5
NRXN1	Neurexin 1
OR52E4	Olfactory Receptor Family 52 Subfamily E Member 4
P38 MAPK	P38 Mitogen-Activated Protein Kinase
p70S6K	p70 Ribosomal Protein S6 Kinase 1
PEG3	Paternally Expressed 3
PIKO	Pacific Innovations, Knowledge, and Opportunities
PNN	Pinin, Desmosome-Associated Protein
PSG	Pregnancy-Specific Glycoproteins
PTPN12	Protein Tyrosine Phosphatase Non-Receptor Type 12
RAP2C-AS1	RAP2C Antisense RNA 1
RGS20	Regulator of G Protein Signaling 20
RIN	RNA Integrity number
RPL/RPS	Ribosomal Proteins
RPL29	Ribosomal Protein L29
RPL39	Ribosomal Protein L39
RPS27A	Ribosomal Protein S27a
RPSA	Ribosomal Protein SA
S100A1	S100 Calcium-Binding Protein A1
SEC63	SEC63 Protein Translocation Regulator
SERPINB7	Serpin Family B Member 7
SKP1	S-Phase Kinase-Associated Protein 1
SLC25A3	Solute Carrier Family 25 Member 3
TAX1BP1-AS1	Tax1-Binding Protein 1 Antisense RNA 1
THBS3-A1	Thrombospondin 3 Antisense RNA 1
TMEM230	Transmembrane Protein 230
UNC5D	Unc-5 Netrin Receptor D
UNC80	Unc-80 Subunit Of NALCN Channel Complex
