# Supplementary material for: Transcriptomic Reprogramming of the Human Placenta Following Maternal Opioid Exposure: Identification of Altered Metabolic and Translational Pathways
Source: Curr Issues Mol Biol. 2026 Sep 3;48(9):900. doi: 10.3390/cimb48090900 (PMC13605290; doi:10.3390/cimb48090900)

Figure S[1]: Distribution of The Level of Gene Expression for The Top Differentially Expressed Genes. Heatmap displaying the log2-transformed normalized counts (via DESeq2 [vst/rlog] transformation) for the top 50 up-regulated (A) and top 50 down-regulated (B) genes identified in Primary Figure 2. This representation illustrates the absolute magnitude of transcript abundance across the 12 samples (n=6, per group). Red indicates high expression and Blue indicates low expression.

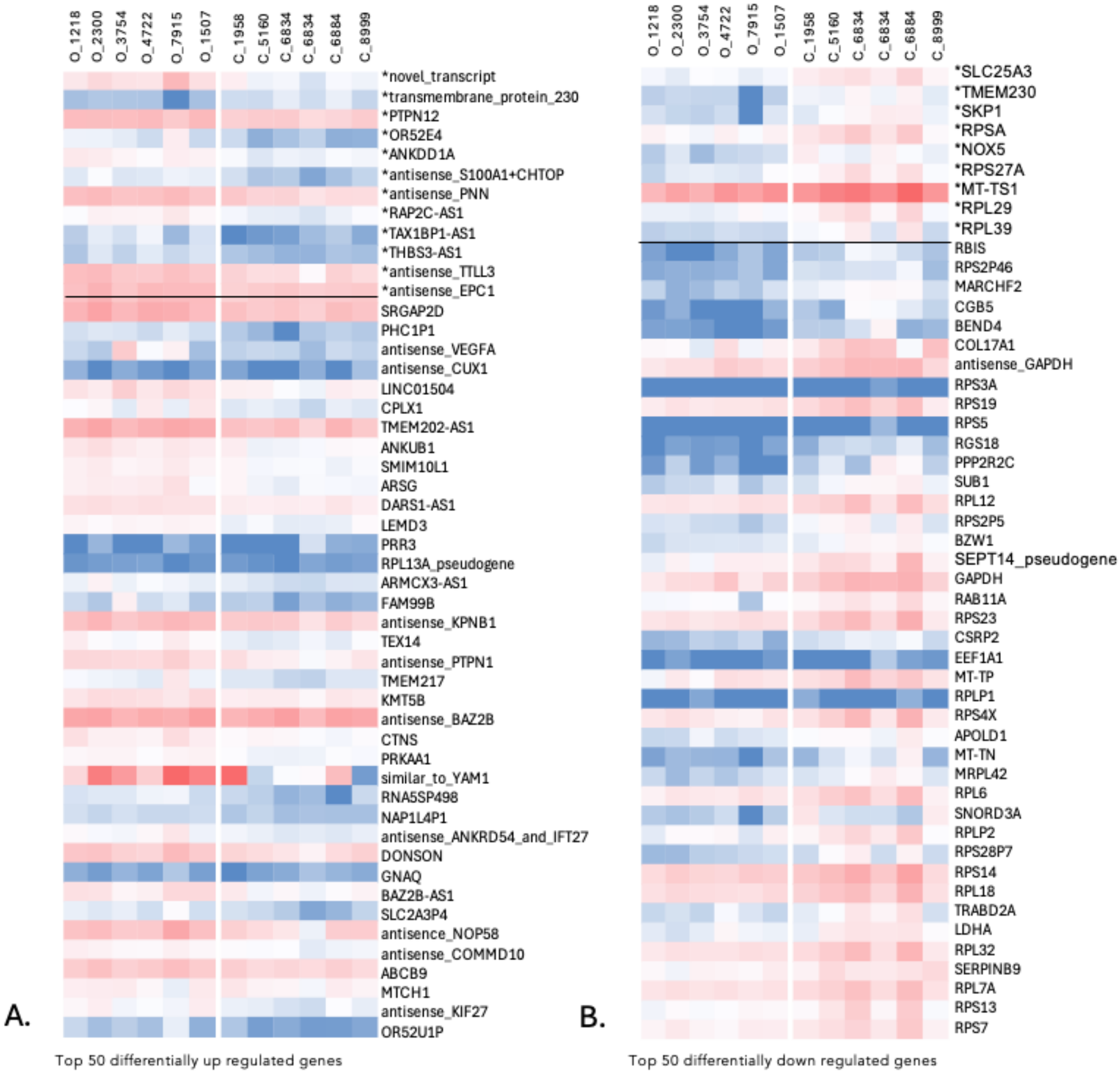

Supplement: Supplementary file 1 [file cimb-48-00900-s001.zip › cimb-4473946-figure S1.pdf]
